# Is there a lower visual field advantage for object affordances? A registered report

**DOI:** 10.1177/17470218241230812

**Published:** 2024-02-18

**Authors:** Annie Warman, Allan Clark, George L Malcolm, Maximillian Havekost, Stéphanie Rossit

**Affiliations:** 1 School of Psychology, University of East Anglia, Norwich, UK; 2 Norwich Medical School, University of East Anglia, Norwich, UK

**Keywords:** Perception, affordances, visual field, grasping, reaction time, hand

## Abstract

It’s been repeatedly shown that pictures of graspable objects can facilitate visual processing, even in the absence of reach-to-grasp actions, an effect often attributed to the concept of affordances. A classic demonstration of this is the handle compatibility effect, characterised by faster reaction times when the orientation of a graspable object’s handle is compatible with the hand used to respond, even when the handle orientation is task-irrelevant. Nevertheless, it is debated whether the speeded reaction times are a result of affordances or spatial compatibility. First, we investigated whether we could replicate the handle compatibility effect while controlling for spatial compatibility. Participants (*N* = 68) responded with left or right-handed keypresses to whether the object was upright or inverted and, in separate blocks, whether the object was red or green. We failed to replicate the handle compatibility effect, with no significant difference between compatible and incompatible conditions, in both tasks. Second, we investigated whether there is a lower visual field (VF) advantage for the handle compatibility effect in line with what has been found for hand actions. A further 68 participants responded to object orientation presented either in the upper or lower VF. A significant handle compatibility effect was observed in the lower VF, but not the upper VF. This suggests that there is a lower VF advantage for affordances, possibly as the lower VF is where our actions most frequently occur. However, future studies should explore the impact of eye movements on the handle compatibility effect and tool affordances.

## Introduction

In everyday life, we are surrounded by thousands of objects that afford different types of interaction. For example, a spoon affords grasping, whereas a bed might afford lying. According to one of the most influential models of perception, when we look at an object, we not only process its colour, shape, and size but we also automatically perceive the potential action it affords, even before we act (Gibson, 1979). Much of the behavioural evidence expanding on Gibson’s concept of affordance stems from the classic Tucker and Ellis (1998) handle compatibility effect characterised by faster reaction times (RTs) when the handle orientation of graspable objects is compatible with the hand used to respond, even when handle orientation is task-irrelevant (see also Tucker & Ellis, 2001, 2004). Tucker and Ellis (1998) attributed the speeded RT for compatible conditions to an automatic triggering of a motor representation afforded by the object’s handle (such as reach-to-grasp) and thus refers to a more representational account of affordances.

In line with this, several neuroimaging studies have reported that simply viewing graspable objects activates sensorimotor brain regions typically associated with reaching, grasping, and using objects (e.g., Chao & Martin, 2000; Creem-Regehr & Lee, 2005). In fact, we have recently reported that hand-selective visual areas in occipito-temporal and parietal cortices automatically encode how to grasp tools correctly for use (i.e., by their handles), even in the absence of subsequent tool use (Knights et al., 2021).

Although the Tucker and Ellis (1998) handle compatibility effect has been widely replicated using various task manipulations (Cho & Proctor, 2010; Pappas, 2014; Saccone et al., 2016; Tipper et al., 2006), whether it is solely explained by affordances remains a subject of controversy. Evidence has shown that compatibility effects are driven by spatial compatibility, and it has been argued that spatial compatibility, rather than object affordances, explains the handle compatibility effect (Cho & Proctor, 2010, 2011; Proctor et al., 2017; see Azaad et al., 2019, for a review). Specifically, a well-known finding is that RTs are faster when the relative spatial location of a stimulus is compatible with the location of the response (e.g., stimulus and response locations are both on the left) even when spatial location is task-irrelevant, a phenomenon known as the Simon effect (Simon, 1969). For example, if the handle of a graspable object (e.g., frying pan) protrudes into the right side of space, the right-hand RTs will be faster due to spatial compatibility between stimulus and response, rather than affordances alone.

In line with the spatial compatibility view, studies have found that the handle compatibility effect is affected by how object stimuli are centred. Specifically, when stimuli are centred with respect to their base or pixel area (thus handles protrude further to one side) the handle compatibility effects are larger compared to when stimuli are simply centred by their width (Kostov & Janyan, 2020; Proctor et al., 2017). Moreover, Cho and Proctor (2011) conducted a study where participants responded to upright and inverted teapot silhouettes and reported compatibility effects towards the spout, rather than handle, of the teapots as the spout protruded further towards the response location. Despite this, others have argued that the outer shape of an object alone (such as a silhouette) may not be sufficient to elicit affordances. For example, Pappas (2014) found compatibility effects for silhouettes both when judgements were made using two fingers within one hand (within-hand) or separate hands (between-hands) and attributed this to spatial compatibility. However, when participants responded either with two fingers of the same hand or separate hands to photographs, the handle compatibility effect only arose when participants responded with separate hands, indicative of an affordance effect. Pappas (2014) therefore suggested that depth information was critical to eliciting the affordance effects, although this inference has recently been the subject of controversy given the differing distance between response keys when participants responded with one hand to when they responded with both hands (Bub et al., 2021).

To dissociate affordances from Simon effects, several manipulations have been added to the Tucker and Ellis’ (1998) upright vs inverted judgement task, such as colour judgements. The idea here is that successful performance on upright vs inverted judgements is considered to elicit affordances, whereas colour judgements depend solely on low-level visual processing, thus not requiring object recognition or affordances (Saccone et al., 2016; Symes et al., 2005). In line with this, it has been shown that handle compatibility effects are larger for judgements of upright vs inverted, semantic categorisation or object shape than for colour judgements (Saccone et al., 2016; Symes et al., 2005; Tipper et al., 2006). This demonstrates that spatial compatibility does not fully contribute to handle compatibility effects, highlighting a likely role of affordances. Nevertheless, this stance remains debatable given that differences in handle compatibility effects between shape and colour judgement tasks have not been replicated (Cho & Proctor, 2012). Therefore, more research is needed to resolve the controversy surrounding the contribution of affordances to the handle compatibility effect.

Another manipulation used to investigate affordances is reaching distance. Several studies have reported that the handle compatibility effect is smaller, or even eliminated, when objects are presented in far (out of reach), as opposed to near (within-reach) space (Ambrosini & Costantini, 2013; Costantini et al., 2010, 2011). Moreover, Saccone and colleagues (2018) did not find a difference between near and far objects when “far” stimuli were still within reach. These findings suggest that the handle compatibility effect depends on an individual’s ability to interact with objects.

Interestingly, to the best of our knowledge, object position in the upper vs lower visual field (VF) has never been compared during affordance tasks. This is important because humans are more efficient at reaching and grasping stimuli presented in the lower VF than in the upper VF, suggesting a functional advantage for the lower VF in visuomotor control (e.g., Brown et al., 2005; Danckert & Goodale, 2001; Krigolson & Heath, 2006). At an anatomical level, several brain areas involved in visuomotor processing (such as V6 and V6A) over-represent the lower VF in both macaques and humans (Galletti et al., 1999; Gamberini et al., 2011; Pitzalis et al., 2010). In fact, we and others have found that visuomotor brain areas (along the medial surface of the parieto-occipital cortex) were significantly more activated when participants reached and grasped objects presented in the lower VF relative to the upper VF (Maltempo et al., 2021; Rossit et al., 2013). Altogether, these findings are consistent with the proposed specialisation of the lower VF for analysis and execution of visuomotor responses (such as grasping and tool manipulation) within peri-personal space (Danckert & Goodale, 2003; Previc, 1990). Thus, it seems reasonable to hypothesise that the VF in which graspable objects are presented may also modulate handle compatibility effects, but this has yet to be investigated.

Therefore, we ran a detailed investigation of handle compatibility effects as well as investigating the effect of the VF in two well-powered pre-registered studies. In Experiment 1, we contrasted upright vs inverted and colour judgements to separate the contribution of Simon and affordance effects and address the debate in the field. We expected to observe larger handle compatibility effects for upright vs inverted judgements than the colour task (e.g., Saccone et al., 2016) which would suggest that affordances contribute to the effects observed. The second experiment investigated, for the first time, whether the handle compatibility effect varies between the upper and lower VFs. Specifically, participants were asked to perform upright vs inverted judgements while fixating on one of two fixation positions allowing objects to be presented in the upper or lower VF. Crucially, by manipulating fixation position rather than the position of the objects, the proximity between stimuli and hands did not differ across conditions. Given the evidence supporting a lower VF advantage for action (e.g., Previc, 1990; Rossit et al., 2013), we hypothesised that the handle compatibility effect would be larger in the lower VF compared to the upper VF.

To our knowledge, this is the first registered report to assess the contribution of affordances to the handle compatibility effect while controlling for spatial compatibility. Some research favouring an affordance account has been subject to failed replications (e.g., Cho & Proctor, 2012; Marshall, 2016 cited in Bub et al., 2018); however, it is possible that these replication studies were underpowered, or the original studies did not provide enough transparency to allow a full replication. Moreover, much of the object-based stimulus-response compatibility (SRC) paradigm literature has employed different methods, for example, number of stimuli, design differences, judgement tasks, sample size justification (or lack of), different exclusion criteria, outlier detection, and analyses. This highlights the importance of pre-registering our methods and analysis plans and using a well-powered design. By including our novel experiment manipulating VF, our entire study design is fully reproducible and replicable to allow researchers to build on the experiment’s findings in the future.

## Methods

### Power analysis

An a priori power analysis was performed using *MorePower 6.0.4* (Campbell & Thompson, 2012) to determine the sample size required. As we were looking for a specific interaction between our independent variable (task or VF) and handle compatibility, we performed our power analysis based on the interaction reported by Pappas (2014; Experiment 2: η_p_^2^ = .143). Pappas’ (2014) Experiment 2 used a 2 × 2 within-subjects design manipulating handle compatibility and, to separate affordances from spatial compatibility, response mode (within- vs between-hands) which closely reflects our experimental design. The power analysis revealed that a sample size of 66 was required to detect a task × compatibility or VF × compatibility interaction with 90% power and α = .05. To allow for equal counterbalancing of blocks, we chose a sample size of 68 for each experiment.

### Participants

Participants were recruited through the University of East Anglia undergraduate participant pool and given course credits for their participation. Each participant was required to only take part in one of the two studies. All participants were aged between 18 and 50. Participants who reported colour blindness, history of neurological disease, motor impairments, or coordination disorders (e.g., dyspraxia) were excluded from the study. Participants were also excluded from analysis if they failed to complete the entire experiment. Excluded participants were replaced until the desired sample size was obtained. All participants provided informed consent in line with the protocol approved by the University of East Anglia School of Psychology Ethics Committee.

### Stimuli and piloting

Stimuli were photographs of common household objects with handles affording a unimanual grasp, presented on a white background. Exemplars were identified from a normative dataset of 296 images extracted from the Bank of Standardized Stimuli (Brodeur et al., 2010; Lagacé et al., 2013) of which 91 exemplars were identified as having handles affording a unimanual grasp. Of the remaining exemplars, 47 were excluded due to not having a clear upright-inverted orientation (e.g., a whisk or potato masher has no clear upright orientation when lying horizontally on a table), and duplicates were removed. Thus, fresh images of the 43 object exemplars were photographed using a Nikon D60 camera, fixed onto a tripod slightly above the object, at 52 centimetres distance to provide depth perspective. Objects were photographed in their upright and inverted orientations with handles oriented to the right, 45° towards the camera. Photographs were cropped to exclude the background and flipped horizontally to create symmetrical left-oriented handled objects. All objects therefore appeared in two horizontal (left, right) and two vertical orientations (upright, inverted), resulting in four unique stimuli for each object. All stimuli were black and white for upright vs inverted judgements (Tucker & Ellis, 1998) and coloured red and green for colour judgements (Saccone et al., 2016). Images were resized so that all objects had the same height, while maintaining aspect ratio, and centred on a transparent background. Because we compared across tasks and VF, we chose to centre objects by their width, rather than base or pixel area (e.g., Cho & Proctor, 2010; Pappas, 2014), as any effects due to object centring will be constant across tasks. Importantly, although the depth cues varied across the vertical axis—upright versus inverted, these cues remained constant across tasks.

To ensure that the vertical orientation of our objects and their names were easily identifiable, we ran a small pilot study to select the final stimulus set. Ten participants were presented with the objects in all four orientations for 100 ms at the fovea. Participants were asked to name the object, identify whether it was upright or inverted, and specify whether the upright/inverted judgement was easy or a guess. Based on the results, we removed 16 objects as the upright/inverted accuracy was less than 90%. A further two objects were excluded as their upright/inverted orientation was guessed by more than 10% of the sample, and one object was excluded because it was incorrectly named by more than 10% of the sample. Where there were multiple exemplars of the same object (e.g., knife, steak knife, cheese knife), we included the exemplar that was most accurately judged without guesses. As a result, 20 objects were selected for the final stimuli set. The number of stimuli closely matched that used in Tucker and Ellis’ (1998) experiment; however, many previous experiments have used a very limited stimulus set (e.g., a single object stimulus; Cho & Proctor, 2011; Tipper et al., 2006). We chose a larger stimulus set to improve ecological validity and include objects with varying handle sizes and orientations to reduce the salience of handle location between trials. All stimuli are available on Open Science Framework https://osf.io/bp8kq/.

### Apparatus

In both experiments, we used an SR Research (Kanata, Ontario, Canada) Eyelink 1000 Plus with a desktop mount system to record participants’ eye gaze and monitor fixation. Monocular vision was recorded at a sampling rate of 500 Hz. Participants sat with their head on a chin rest at a fixed distance of 60 cm from a 24″ BenQ XL2411Z monitor.

## Experiment 1

Our first experiment sought to replicate the handle compatibility effect while controlling for the spatial compatibility which has previously been shown to influence compatibility effects (Cho & Proctor, 2010; Kostov & Janyan, 2020; Proctor et al., 2017). Using a within-participants design, participants responded with their left or right hand to whether the handled object was upright or inverted or, in separate blocks, whether the object was red or green. Unbeknown to participants, handles were oriented towards the same side as the correct response (compatible), or the opposite side (incompatible).

### Participants

In Experiment 1, 68 participants aged between 18 and 46 took part (18 male; *M*_age_ = 20.8, *SD* = 4.32). Participants were recruited through the University of East Anglia undergraduate participant pool and given course credits for their participation. Sixty participants were right-handed, six were left-handed, and two were ambidextrous based on the Edinburgh Handedness Inventory (mean laterality index = 67.4, *SD* = 55.6).

### Procedure

Following informed consent, participants completed a short demographics questionnaire to ensure they fulfilled the inclusion criteria. Following this, participants completed the eye-tracking calibration procedure and the eye with higher spatial accuracy was selected for monocular recording.

In the main handle compatibility task, each trial began with a fixation bullseye (1°) presented at the centre of the screen for a fixed duration of 1,000 ms, followed by a variable delay of 500–1,250 ms (with a random delay of 250 ms intervals). Then, a stimulus (maximum 15° × 5°) appeared in the centre of the screen until a response was made (maximum presentation time = 1,500 ms). In separate blocks, participants were asked to judge either whether the object is normal (upright) or inverted according to its canonical orientation or responded to the colour (red or green) by pressing either “q” with their left index finger or “p” with their right index finger on a QWERTY keyboard as quickly as possible. Note here that the task instructions used the term “normal” instead of “upright” to prevent any response advantages due to the lexical similarity between “upright” and objects presented in the “upper” VF in our second experiment (Saccone et al., 2016). In addition, participants were required to maintain fixation throughout the trial. Feedback was provided reiterating the required response buttons in the event of an inaccurate response or when the response was not initiated within 1,500 ms. Participants were also informed of eye movement errors in the event of fixation errors greater than 1.5°. In the event of multiple consecutive eye movement errors due to calibration failure, a recalibration procedure took place, and if necessary, the selected eye was changed to the eye with higher spatial accuracy.

**Figure 1.**
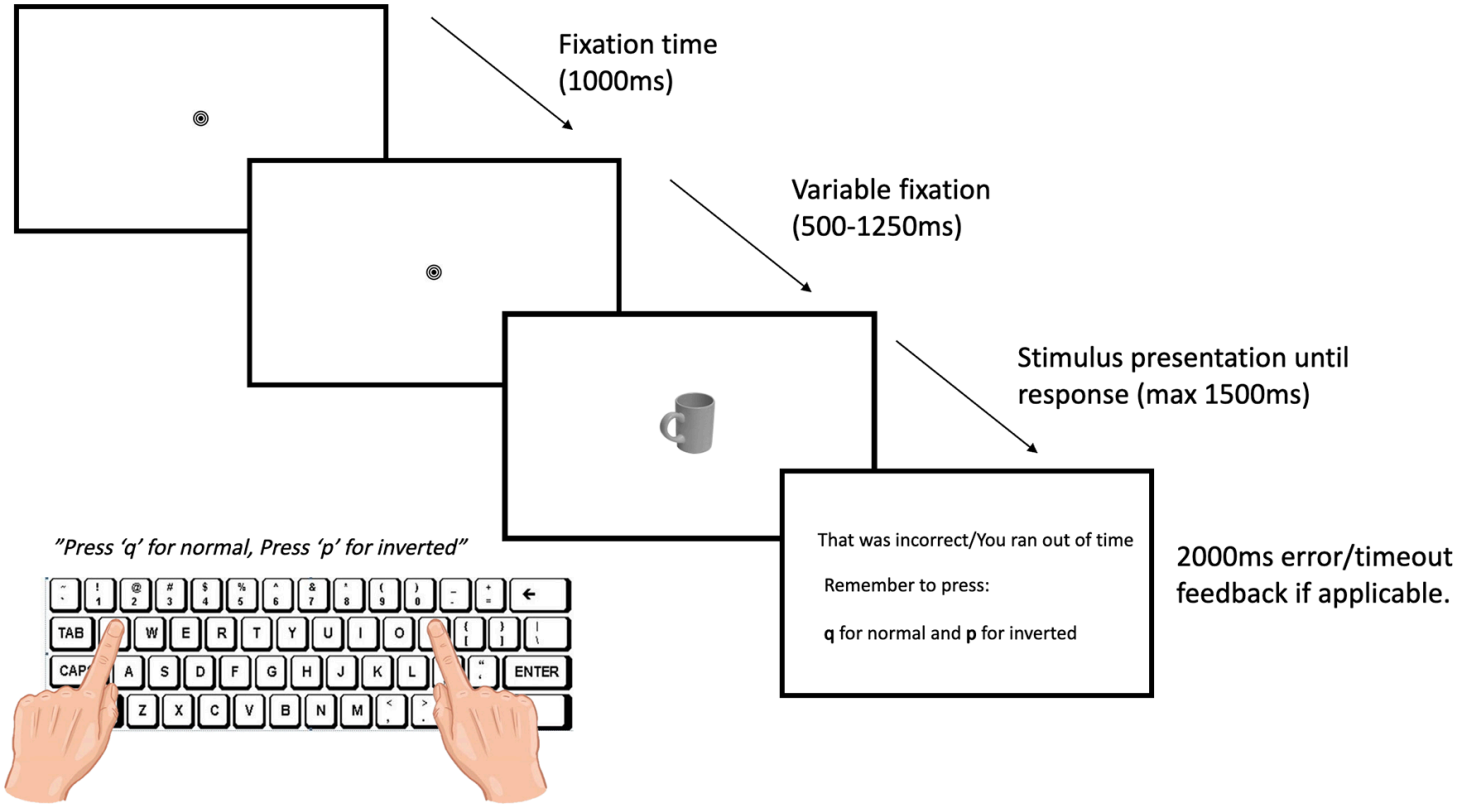
Timing and sequence for Experiment 1 with an example of a compatible trial.

In a compatible trial, the hand used to respond was congruent with the orientation of the object handle, whereas for incompatible trials the hand used to respond, and the handle orientation was incongruent (see Figure 1). Response mapping was counterbalanced across blocks.

The experiment consisted of four blocks: in two consecutive blocks, participants performed upright vs inverted judgements, and in the other two blocks, participants performed colour judgements. Block order was counterbalanced so that half of the participants began by judging object orientation (upright/inverted) and the other half began by judging colour. In each block, each stimulus was randomly presented once in each possible horizontal handle orientation (left, right) and vertical orientation (upright, inverted), resulting in 20 × 2 × 2 = 80 trials per block, and a total of 320 trials in the entire experiment. There were an equal number of compatible and incompatible trials per block. Each block commenced with 16 practice trials (2 independent stimuli × 2 handle orientations × 2 vertical orientations × 2 repetitions). Practice stimuli were independent exemplars that were excluded based on the pilot study. Participants initiated each block by pressing the spacebar and took a break between each block for a minimum of 20 s to reduce fatigue and eye discomfort. The experiment was developed using Experiment Builder (SR Research).

### Data analysis

Trials in which participants did not respond within 1,500 ms, responded incorrectly, or a fixation error of greater than 1.5° was detected, were excluded from all analyses. For a participant to be included in the final analysis, a minimum of 20 correct trials per condition was needed to compute a mean. Participants excluded at the data analysis stage were replaced until the sample size of 68 was achieved. For each participant, the mean RT for each condition (task: upright/inverted, colour; handle: compatible, incompatible) was calculated. RTs greater than two standard deviations away from each participant’s condition mean were excluded as outliers (Pappas & Mack, 2008; Symes et al., 2005). A 2 (task: upright/inverted, colour) × 2 (handle: compatible, incompatible) repeated-measures analysis of variance (ANOVA) was conducted on mean RTs with Bonferroni-corrected post hoc comparisons. In the event of null effects, non-rejection of the null hypothesis was clarified by using two one-sided tests (TOST) (Lakens, 2018) giving a *p*-value which is the larger of the two one-sided *p*-values testing the null hypothesis that the effects were less than (in numerical value) that deemed to be minimally important. The smallest effect size of interest (SESOI) was set at d*z* = 0.106 for all TOST calculations, and this is the effect size reported for the handle compatibility effect in a recent meta-analysis (Azaad et al., 2019). This was a deviation from the preregistration in which we specified the SESOI would be derived from the sample size calculation. We were unable to calculate the dz from the Pappas (2014) paper due to the lack of open data to calculate the correlation between the within-subjects measures. Moreover, the effect sizes reported would have been considerably larger than those reported in the meta-analysis, thus we adopted a more conservative approach. Although using the lower bound estimate could deal with the bias reported in the meta-analysis, given the overall average effect size was small (10 ms), we deemed this is the most appropriate SESOI to use.

Data analysis was performed in R version 4.1.3, using the *tidyverse* (version 1.3.1), *rstatix* (version 0.7.0), and *TOSTER* (version 0.7.1) packages. Analysis code is available on OSF https://osf.io/bp8kq/.

## Results

A total of 4,713 (21.7%) trials were excluded from the main analysis. This included trials where participants made eye movement errors (11.2% of total trials), incorrect responses (6.3%), time outs (0.4%), and where RT was greater than two standard deviations from the participant’s condition mean (3.8%).

### Reaction time

**Figure 2.**
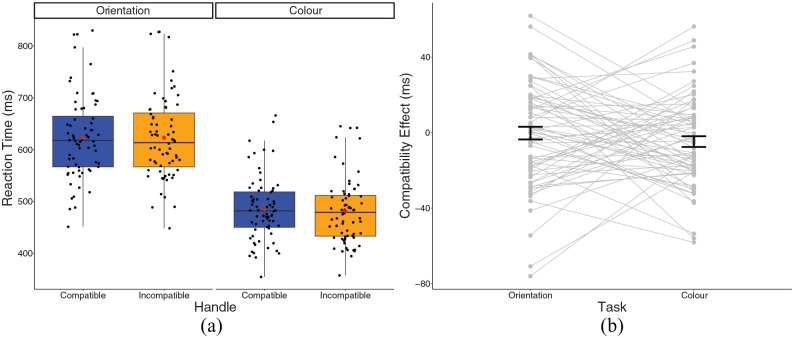
(a) A box plot displaying reaction times for Experiment 1 in the experimental conditions. Black dots represent individual data points, red dot represents the condition mean. (b) A plot displaying the compatibility effect in the experimental conditions. Dots and lines represent individual data points, error bar represents standard error around the mean.

A 2 × 2 repeated-measures ANOVA revealed a main effect of task, *F*(1, 67) = 267.95, *p* < .001, η_p_^2^ = .800. Unsurprisingly, RTs in the colour task (*M* = 483.71, *SD* = 62.58) were significantly faster than the orientation task, *M* = 622.98, *SD* = 80.45, *t*(67) = 16.36, *p* < .001, see Figure 2. There was no significant effect of compatibility, or task × compatibility interaction.

The TOST procedure revealed that the compatibility effect for orientation judgements was smaller than the SESOI, *t*(67) = 2.48, *p* = .008. RTs in the incompatible condition (*M* = 622.86, *SD* = 80.86) were similar to the compatible condition (*M* = 623.09, *SD* = 80.65). However, the compatibility effect for colour judgements was not statistically equivalent. We failed to reject the hypothesis that the true compatibility effect size for the colour task was at least as large as the SESOI, 0.106; *t*(67) = 0.68, *p* = .249, although this was in the direction of a negative compatibility effect as RTs in the incompatible colour condition (*M* = 481.35, *SD* = 63.27) were slightly faster than in the compatible colour condition (*M* = 486.07, *SD* = 62.26).

## Experiment 2

In Experiment 2, we investigated whether the handle compatibility effect is larger in the lower VF, given the lower VF advantage in visuomotor control (Rossit et al., 2013). Participants responded with the left or right hand depending on whether the object was upright or inverted to stimuli presented in the upper or lower VFs. Crucially, to control for hand-object proximity effects, only the fixation position was manipulated, and all stimuli were presented centrally. As in Experiment 1, the object’s handle was either compatible or incompatible, with the hand used to correctly respond.

### Participants

In Experiment 2, 68 participants aged between 18 and 44 took part (14 male, 1 non-binary; *M_age_* = 21.8, *SD* = 5.87). Participants were recruited through the University of East Anglia undergraduate participant pool and given course credits for their participation. Fifty-four participants were right-handed, eight were left-handed, and six were ambidextrous based on the Edinburgh Handedness Inventory (mean laterality index = 62.7, *SD* = 58.0).

### Procedure

**Figure 3.**
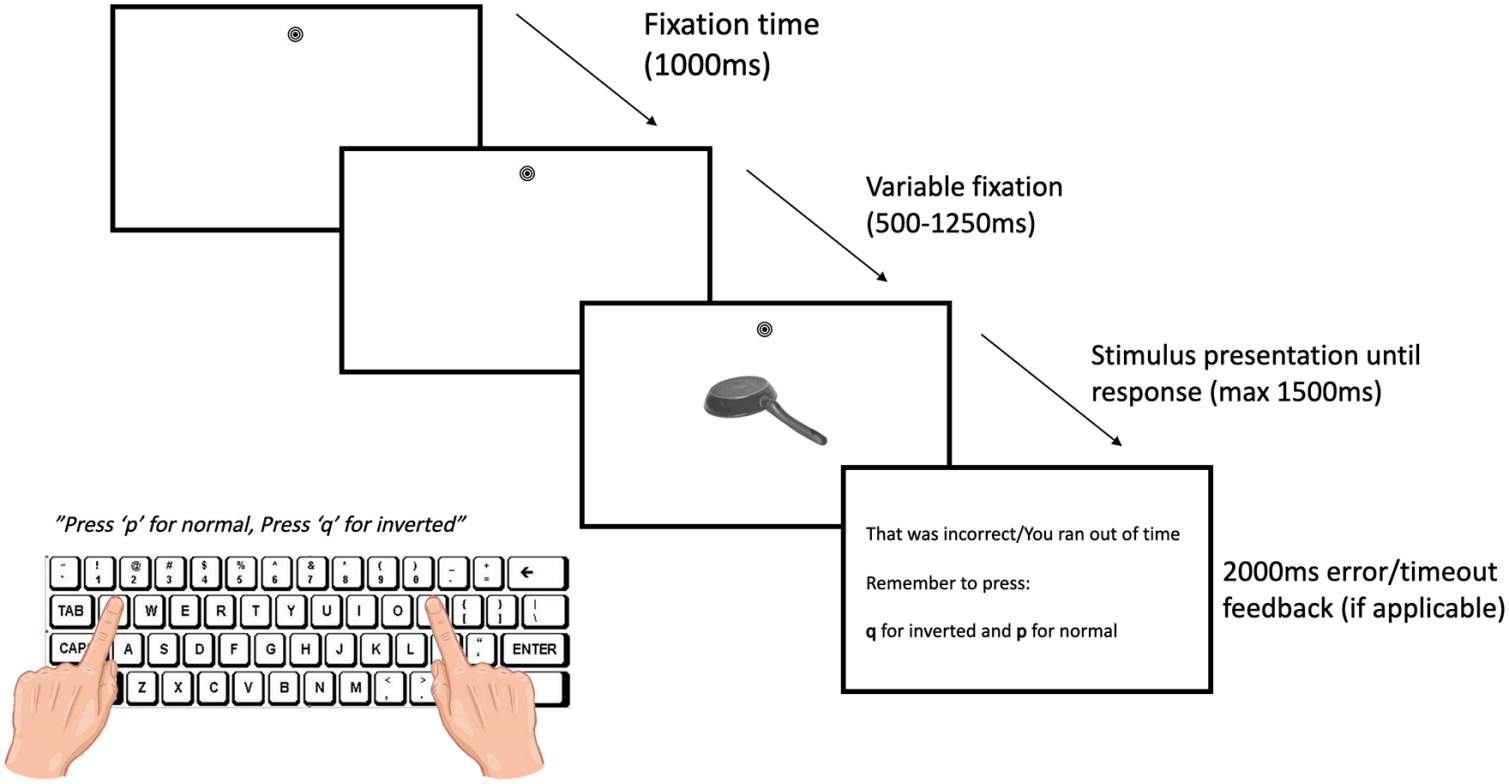
Timing and sequence for Experiment 2 with an example of an incompatible trial in the lower VF.

The procedure for Experiment 2 remained the same as Experiment 1. However, in the handle compatibility task, participants only performed upright vs inverted judgements and not colour judgements. In a typical trial, the fixation bullseye was randomly presented either 7° above or below the centrally presented object and remained on screen throughout each trial. The next trial began with the fixation bullseye presented for 1,000 ms to allow participants to fixate, following which there was a variable delay period as in Experiment 1 (see Figure 3).

### Data analysis

Data exclusion criteria remained the same as in Experiment 1. A 2 (handle: compatible, incompatible) × 2 (VF: upper, lower) repeated-measures ANOVA was conducted on mean RTs, and post-hoc comparisons were Bonferroni corrected. Null effects were followed up with the TOST procedure with the SESOI set to 0.106 (Lakens, 2018).

## Results

A total of 6,914 (31.8%) trials were excluded from the main RT analysis. These included trials where participants made eye movement errors (12.8%), responded incorrectly (14.9%), timed out (0.5%), or responded more than two standard deviations away from the condition mean (3.5%).

### Reaction time

**Figure 4.**
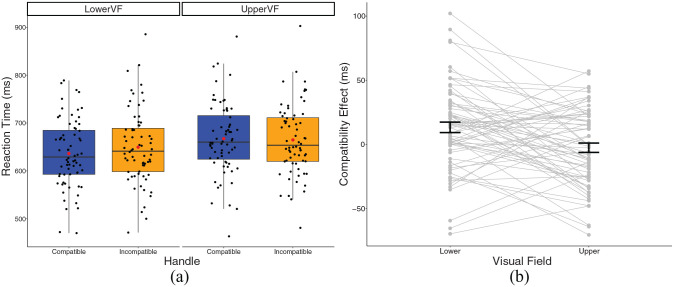
(a) A box plot displaying reaction times for Experiment 2 in the experimental conditions. Black dots represent individual data points, red dot represents the condition mean. (b) A plot displaying the compatibility effect in Experiment 2 experimental conditions. Dots and lines represent individual data points, and error bar represents standard error around the mean.

A 2 × 2 repeated-measures ANOVA revealed there was a significant main effect of VF, *F*(1,67) = 36.82, *p* < .001, η_p_^2^ = .355, which was qualified by a significant compatibility by VF interaction *F*(1, 67) = 11.26, *p* = .001, η_p_^2^ = .144. As hypothesised, we found a significant handle compatibility effect in the lower VF only: RTs in the incompatible condition (*M* *=* 649.52, *SD* = 77.32) were significantly higher than RTs in the compatible condition (*M* = 636.27, *SD* = 70.87), *t*(67) = 3.25, *p* = .002. On the contrary, in the upper VF, there was no significant difference between RTs in the incompatible condition (*M* = 664.65, *SD* = 73.05), and RTs in the compatible condition (*M* = 667.29, *SD* = 74.58), *t*(67) = 0.726, *p* = .470 (see Figure 4). The TOST was also non-significant, *t*(67) = 1.42, *p* = .080, thus we cannot reject a true effect at least as large, or larger, than the SESOI of 0.106, although this was in the direction of a negative compatibility effect.

### Secondary analyses (all studies)

Although some studies report handle compatibility effects on error rates, for example higher errors in incompatible conditions (Pappas, 2014; Tucker & Ellis, 1998), other reports have not replicated this (Goslin et al., 2012; Saccone et al., 2016). To clarify this, we explored the effect of handle-response compatibility on error rates in each experiment. In Experiment 1, a 2 (handle compatibility: compatible, incompatible) × 2 (task: orientation, colour) repeated-measures ANOVA was conducted on percentage error (PE). In Experiment 2, a 2 (handle compatibility: compatible, incompatible) × 2 (VF: lower VF, upper VF) ANOVA was conducted on PE. Post hoc comparisons were Bonferroni-corrected, and null effects followed up using TOST (Lakens, 2018). All secondary analyses were planned prior to data collection and included in the Stage 1 report.

### Percentage error—Experiment 1

In the orientation task, there was an average error rate of 9.04% (*SD* = 5.47) in the compatible condition and 9.65% (*SD* = 6.21) in the incompatible condition. There was higher accuracy in the colour tasks, with an average error rate of 3.49% (*SD* = 2.89) in the compatible condition and 2.90% (*SD* = 2.56) in the incompatible condition.

**Figure 5.**
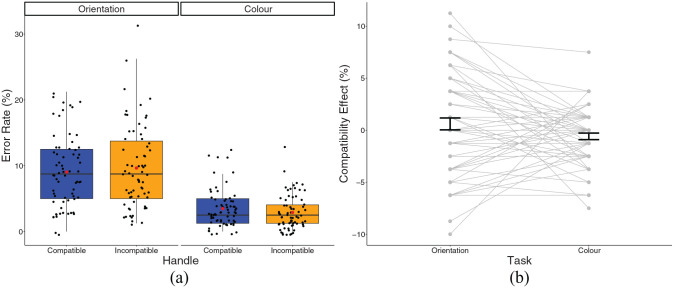
(a) A box plot displaying percentage error in the experimental conditions in Experiment 1. Black dots represent individual data points, red dot represents the condition mean. (b) A plot displaying the compatibility effect in the experimental conditions. Dots and lines represent individual data points, error bar represents standard error around the mean.

In line with the findings from our RT analysis, a 2 × 2 repeated-measures ANOVA revealed a significant main effect of task *F*(1,67) = 114.2, *p* < .001, η_p_^2^ = .630, with significantly higher error rates in the orientation task compared to the colour task *t*(67) = 10.69, *p* < .001. There was no effect of compatibility or interaction (see Figure 5).

The TOST procedure failed to provide evidence of statistical equivalence for the compatibility effect in the orientation condition, *t*(67) = 0.03, *p* = .487, or in the colour condition, *t*(67) = 0.946, *p* = .826.

### Experiment 2

Error rates were slightly higher in Experiment 2, possibly due to all tasks involving orientation judgements. In the lower VF, there was an average error rate of 13.62% (*SD* = 7.30) in the compatible condition and 16.12% (*SD* = 6.26) in the incompatible condition. In the upper VF, there was an average error rate of 15.79% (*SD* = 6.35) in the compatible condition and 14.43% *(SD* = 6.66) in the incompatible condition.

**Figure 6.**
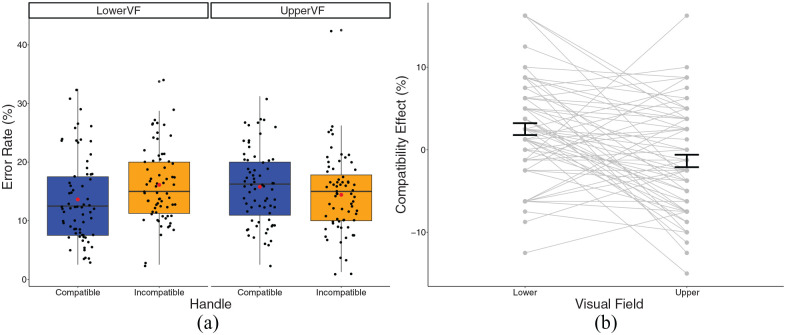
(a) A box plot displaying the percentage error in Experiment 2. Black dots represent individual data points, red dot represents the condition mean. (b) A plot displaying the compatibility effect in the Experiment 2 experimental conditions. Dots and lines represent individual data points, error bar represents standard error around the mean.

A 2 × 2 repeated-measures ANOVA found a significant VF by compatibility interaction, *F*(1, 67) = 17.07, *p* < .001, η_p_^2^ = .203 (see Figure 6). Bonferroni-corrected post-hoc *t*-tests revealed a significant compatibility effect in the lower VF, significantly more errors were made in the incompatible condition, compared to the compatible condition, *t*(67) = 3.47, *p* < .001; however, there was no significant compatibility effect in the upper VF, *t*(67) = 1.80, *p* = .076. The TOST procedure failed to provide evidence of statistical equivalence for the compatibility effect in the UVF, *t*(67) = 0.89, *p* = .812; however, this was in the direction of a negative compatibility effect.

Moreover, accuracy was significantly better in the lower VF than the upper VF, in the compatible condition, *t*(67) = 3.23, *p* = .002. However, in the incompatible condition, accuracy was significantly worse in the lower VF, compared to the upper VF, *t*(67) = 2.57, *p* = .012. There was no significant main effect of compatibility or VF.

### Exploratory analyses

To investigate the temporal development of handle effects in each condition, we conducted a distribution analysis planned in advance of data collection. Participants’ correct RTs for compatible and incompatible trials were rank-ordered, divided into four equal bins, and the mean RT for compatible and incompatible trials in each bin was calculated. A handle compatibility effect was then calculated for each bin by subtracting the mean RT for compatible trials from the RT for incompatible trials, resulting in quartile effect sizes from Experiment 1 for both the upright/inverted judgement task and the colour judgement task. Here, we expected that the handle compatibility effect would emerge over time when participants judge whether objects are upright/inverted. This pattern would be consistent with previous findings in distribution analyses for handle compatibility effects when tasks require object recognition (e.g., upright/inverted, kitchen/shed; Saccone et al., 2016; Symes et al., 2005). When the task is to judge colour, however, we expected the handle compatibility effect to be present in the earlier quartiles, but to rapidly dissipate in the latter quartiles, consistent with the literature on the temporal profile of the Simon effect (De Jong et al., 1994; Proctor et al., 2011). We had no predictions for the time course of effects in the upper and lower VFs. To statistically assess any effects across the RT distribution, participants’ mean effect sizes for each bin and condition were entered into two 4 (bin: 1, 2, 3, 4) × 2 (Task or VF) repeated-measures ANOVAs.

### Results

#### Experiment 1

Given that colour judgements were significantly faster than orientation judgements, we conducted a bin analysis to investigate whether there were different mechanisms contributing to the compatibility effect. There was a small negative compatibility effect across all four time bins in the colour task. In the orientation task, a small negative compatibility effect was observed in the earliest time bin, however as RT increased, the compatibility effect increased slightly (see Table 1).

**Table 1.** Mean compatibility effects across the four time bins for both the colour and orientation tasks in Experiment 1.

Bin	Compatibility effect (ms)	Colour task
	Orientation task	
1	−7.80 (22.4)	−6.41 (19.4)
2	0.98 (23.7)	−5.23 (23.1)
3	4.76 (35.3)	−2.25 (29.9)
4	2.17 (52.0)	−4.19 (43.7)

*Note*. Numbers show mean. *SD*: standard deviation.

A 4 × 2 repeated-measures ANOVA revealed a small but significant effect of bin *F*(1.77, 118.55) = 3.21, *p* = .05, η_p_^2^ = .046. Post-hoc comparisons revealed that there was a significantly larger compatibility effect in bin 2 compared to bin 1, *t*(67) = 2.80, *p* = 007, and in bin 3 compared to bin 1, *t*(67) = 3.08, *p* = .003. There was, however, no effect of task or interaction.

#### Experiment 2

Our bin analysis for Experiment 2 revealed a main effect of VF, *F*(1, 67) = 8.26, *p* = .005, η_p_^2^ = .110, where there was a significantly higher compatibility effect in the lower VF (*M* = 11.94, *SD* = 54.87) compared to the upper VF (*M* = -2.78, *SD* = 55.14), *t*(67) = 2.87, *p* = .005. There was no effect of bin or interaction (see Table 2).

**Table 2.** Mean compatibility effects per bin in the lower and upper VFs in Experiment 2.

Bin	Compatibility effect (ms)	
	Lower VF	Upper VF
1	9.16 (61.0)	−2.15 (53.2)
2	14.22 (46.7)	2.07 (53.9)
3	9.38 (57.5)	−1.85 (57.5)
4	14.99 (54.1)	−9.18 (58.7)

*Note*. Numbers show mean (*SD*). VF: visual field.

## Discussion

The functional specialisation of the lower VF for visuomotor control has been demonstrated by a number of neuroimaging and behavioural studies which have provided evidence for increased speed and accuracy for movements towards targets in the lower VF compared to upper VF (Brown et al., 2005; Danckert & Goodale, 2001; Krigolson & Heath, 2006; Stone et al., 2019), and increased activation in visuomotor brain regions when performing actions in the lower VF (Maltempo et al., 2021; Rossit et al., 2013). Moreover, area V6A in the macaque, which is thought to compute object affordances (Breveglieri et al., 2015), over-represents the lower VF (Galletti et al., 1999). It is logical to assume that humans have developed this functional specialisation given that most of our actions with objects in day-to-day life are performed in the lower VF. Indeed, this has recently been quantified for the first time: over 70% of our actions with objects are performed in the lower VF (Mineiro & Buckingham, 2023).

The findings of our Experiment 2 demonstrate that a lower VF advantage for action, and possibly affordances, is present with images of graspable objects, even when the object orientation is irrelevant to the task goal. First, RTs were faster in the lower, compared to upper, VF, consistent with previous behavioural literature (Brown et al., 2005; Danckert & Goodale, 2001). Second, we observed a significant handle compatibility effect for both RTs and accuracy in the lower, but not upper, VF. However, we failed to replicate the handle compatibility effect in our Experiment 1, where participants were presented with objects in their foveal vision; nor did we observe any differences in the compatibility effect between our two tasks (judging orientation vs colour), except for colour judgements being significantly faster than orientation judgements. Therefore, in conjunction with the findings of Experiment 1, we can only speculate as to the possible explanations for the VF difference in the handle compatibility effect.

Our findings of a lower VF advantage in the handle compatibility effect are in line with previous research demonstrating that compatibility effects are reduced, or eliminated, when objects are presented in extra-personal, as opposed to peri-personal space (Ambrosini & Costantini, 2013; Costantini et al., 2010, 2011). In these experiments, objects in peri-personal space were presented lower on the vertical meridian than those in extra-personal space. In one sophisticated manipulation however, Costantini et al. (2010) presented objects in the same position either in front of, or behind, a clear screen. In a striking case for the affordance account, a handle compatibility effect was only observed when the object was in front of the screen and thus manipulable. Put together, our findings provide complementary evidence for a lower VF advantage in reaching and object manipulation in peri-personal, reachable, space (Previc, 1990). Here, findings apply specifically to the VF of presentation, as we controlled for hand-object proximity by manipulating fixation position, rather than the object position, on the screen.

Given that we manually interact with and use objects mostly in the lower VF, the lower VF compatibility effect may therefore be reflective of activation of action-related information to allow for successful interaction with the object, in line with affordance accounts (Tucker & Ellis, 1998). This explanation seems plausible given all objects were centred on the screen with respect to their width (and thus a reduced salience of the handle towards a single side of space; Azaad & Laham, 2020). Numerous previous keypress response SRC paradigms have reported no compatibility effect, or even negative compatibility effects, when objects are centred by their width (Bub et al., 2021; Kostov & Janyan, 2020; Lien et al., 2014; Yu et al., 2014). These findings have been explained by a spatial account due to the functional end, rather than handle, protruding more to one side, thus facilitating responses compatible with the functional end due to spatial coding. Therefore, a purely spatial account of our findings would predict a negative compatibility effect across all our tasks due to stimuli being centred by their width. Our findings therefore cannot be explained by a purely spatial account given that we failed to observe a negative compatibility effect across any tasks, and a significant compatibility effect was present when stimuli were presented in the lower VF. Despite this, we failed to observe a compatibility effect in Experiment 1 when the task was to judge orientation, and thus thought to elicit affordances, which questions the contribution of affordances to the handle compatibility effect.

It is possible that the lack of compatibility effect observed in Experiment 1 for both the colour and orientation tasks was due to the restriction of eye movements. To our knowledge, this is the first study using the SRC paradigm with handled objects while requiring participants to maintain fixation throughout trials, thus the effects of restricting eye movements remain unknown. A number of eye-tracking studies have demonstrated that visuospatial attention is biased towards the action-performing side of an object, as opposed to the handle (Pilacinski et al., 2021; van der Linden et al., 2015). Moreover, the bias towards the action-performing side of the object has been shown to increase over the time course, suggesting that the action-related effects may be more likely to build up over time and when the object is foveated (van der Linden et al., 2015). This suggests that the eye is driven towards the functional part of the tool, potentially to recognise the tool’s functional use. As participants were required to inhibit eye movements to either side of the object, it is possible that stimuli were harder to recognise, and action-related information was less salient. For instance, by recognising an object by its functional end, one can adjust the grip aperture and posture to successfully use the object (Belardinelli et al., 2016). Our finding of a lack of compatibility effect in Experiment 1, as well as no effect differences across the time course, could therefore be explained by the inhibition of eye movements restricting object identification, thus not eliciting affordances (Saccone et al., 2016; Symes et al., 2005). It is also possible that, given our findings from Experiment 2, affordances are only elicited to objects in the lower VF. For example, it could be speculated that previous failed replications are a result of participants making eye movements towards a keyboard in the lower VF to respond, thus moving the object into the upper VF and reducing the effect. Future studies could employ eye-tracking measures alongside the task to investigate how eye movements modulate RTs in keypress SRC paradigms.

Of course, our failure to replicate the handle compatibility effect in Experiment 1 further questions the reliability of using a keypress handle SRC paradigm as a measure of affordances. Despite this, there remains a growing body of literature providing a motor-based account of compatibility effects. The handle compatibility effect has recently been replicated in both lab-based experiments, and online (Littman et al., 2023; Littman & Kalanthroff, 2022). In both experiments here, however, participants were primed by observing, or engaging in, hand-object interactions with the stimuli used. Moreover, only RTs for upright objects were included in the analysis which may explain the lack of effects observed in our Experiment 1.

In further support for a motor-based account, Zou et al. (2022) observed significant handle compatibility effects when handles were broken following 50 ms of stimulus presentation; however, this disappeared when the handle was broken at a later stage (150 ms, 250 ms). Despite this, a compatibility effect was present when the handle remained intact (and thus graspable), which was not observed with symmetrical objects and when “handles” were protruding shapes. Therefore, it seems likely that both spatial coding and affordances contribute to handle compatibility effects, with affordance-related effects occurring later than spatial effects.

More recent research has shown that compatibility effects also depend on participants’ motor intentions. This has been demonstrated in experiments reporting a negative compatibility effect in keypress response paradigms, but a positive compatibility effect when participants are required to respond with a reach and grasp movement (Bub et al., 2021; Bub & Masson, 2010; Ferguson et al., 2021). These findings suggest that the compatibility effect depends on the action-related information of the task demands, with compatibility effects only arising when participants’ action intentions are to perform a reach-to-grasp movement, rather than a keypress with the index finger. Indeed, we do not typically interact with objects without a reach-to-grasp movement. Future research could assess VF differences in the handle compatibility effect using reach-to-grasp responses. It would also be interesting to assess movement kinematics to investigate which stage of a reach-to-grasp action these compatibility effects arise. It is possible that employing a reach-to-grasp paradigm would reduce the heterogeneity observed in our present experiments and generate more robust findings.

Overall, using a well-powered, and well-controlled experimental design, we failed to replicate the highly cited Tucker and Ellis (1998) handle compatibility effect when participants fixated on the object centre while making keypress responses to the objects’ orientation. Moreover, no compatibility effect was observed when participants responded to object colour. However, a significant compatibility effect and faster responses were observed when objects were in participants’ lower VF. This adds to a body of evidence suggesting a lower VF advantage for action. Although we cannot conclusively explain our findings in terms of a lower VF advantage in affordances, the presence of a compatibility effect in the lower VF cannot be explained by spatial compatibility. Future research should further investigate vertical VF differences in affordances using reach-to-grasp SRC paradigms as task demands will be more relevant for action. Moreover, caution should be used when interpreting handle compatibility effects in keypress SRC paradigms in terms of affordances.
